# Will Technology Rather Than Vaccination Be the Way to Control Pandemics?

**DOI:** 10.1097/IM9.0000000000000041

**Published:** 2020-10-23

**Authors:** Sebastian Leptihn, Susan C. Welburn

**Affiliations:** 1 Zhejiang University-University of Edinburgh (ZJU-UoE) Institute, Zhejiang University, International Campus, Haining, Zhejiang, China; 2 Edinburgh Medical School, Biomedical Sciences, College of Medicine & Veterinary Medicine, The University of Edinburgh, Edinburgh, United Kingdom; 3 Department of Infectious Diseases, Sir Run Run Shaw Hospital, Zhejiang University School of Medicine, Hangzhou, Zhejiang, China.

At policy level for the COVID-19 pandemic two main themes emerge, containment of infection (testing, tracing, and isolation) and vaccination. The pandemic has stimulated rapid development of vaccine candidates that are in various phases of clinical trials, some of which are advanced with regulatory approvals expected early in the new year.^1,2^

However, even with vaccination on the horizon there are multiple constraints to overcome before this pandemic will be consigned to history. The enormous task of vaccine production and distribution, possibly involving repeated vaccinations and management of non-compliance within populations are a major challenge for 21^st^ century Global Public Health policy.

Multiple “waves” of infection are expected to sweep across the globe in the coming winter months, confounded with seasonal flu and other winter viral infections. Once this pandemic is vanquished how do we prepare more effectively for the next? Emerging infections are becoming more and more frequent with increased human encroachment into animal habitats and increased pandemic preparedness is needed.

Communication is critical between governments to develop and adopt synergistic technologies for global prevention and control, to address bottlenecks for essential medicines and equipment and to work together in biosecurity to mitigate risks of emerging infectious diseases, putting in place robust and effective surveillance and control measures.

Smartphone technology is becoming ubiquitous and many countries have developed tracing apps to inform a user of potential exposure to an infected person and to support and in some cases enforce self-isolation to prevent viral spread. However, never had there been such high demand for diagnostic services and test and trace systems in most countries where infection has become systemic are inadequate, slow, and inefficient. In addition, voluntary quarantine is highly problematic in terms of compliance, aside from being highly disruptive to all of society; as recently evidenced by the dramatic recent resurgence of cases across Europe and the US.

One promising approach that offers a potential solution to tackling the pandemic is the development of simple, robust personal testing devices integrated with a smartphone app that could feed into public health data collection systems. Testing for antibodies to determine whether a person had been infected (or is in an advanced stage of infection) may be of limited use for COVID-19 but tests that offer a cheap and quick test for the virus itself would be of value. Tests with single-virus sensitivity using direct, real-time electrical detection,^3^ label-free optical heterodyne detection^4^ or fluorescence,^5^ some compatible for the use with smartphones^6,7^ or hand-held devices based on a Young interferometer sensor device for ultrasensitive detection of viruses,^8^ among other methodologies that detect viral particles or their components^9,10^ may offer a solution. Putting testing in the hands of the individual is now mainstream for management of diabetes; a similar approach may be needed for when SARS-CoV-2 becomes endemic. Futuristic platforms such as the CRISPR-based, smartphone camera-facilitated detection of SARS-CoV-2 RNA with results obtained in minutes offer a technological breakthrough^7^; miniaturized into robust, easy to use devices, mass produced for use as handheld point-of-care instruments.

There will be many arguments against such practice from public health bodies and consumer bodies alike, but in countries where SARS-CoV-2 had become endemic, where test and trace cannot keep up with demand, and where even if a vaccine is available compliance with vaccination policy is low, cheap fast diagnostic tests for personal use will become an essential tool for living with the virus. Who would not want assurance they are COVID-19 free before seeing a patient or before visiting vulnerable individuals? Systems to share personalized data would be of value to national public health agencies, however, individuals may not want to permit access to their health data but solutions that offer security and data protection can be found. As a society we have a duty to protect ourselves and to protect others; this pandemic is likely to be the most serious health crisis we will face in our lifetime. Perhaps it is time to review how public health is and can be managed, accepting that technology offers a significant opportunity for individuals to ultimately take responsibility for their own health during this pandemic and factoring this into public health planning for the next.
